# Controlling the Diffusion Profile in the Vapor Phase Deposition of Silanes for Gradient Fabrication

**DOI:** 10.1021/acs.langmuir.5c03462

**Published:** 2026-03-26

**Authors:** Shomaly Chakraborty, Ash Young, Md. Abdullah Al Macktuf, Sarah C. Rutan, Maryanne M. Collinson

**Affiliations:** Department of Chemistry, 6889 Virginia Commonwealth University, Richmond, Virginia 23284, United States

## Abstract

Surface chemical gradients were prepared on porous supports using a modified vapor deposition approach, and the shape and length of the gradient were controlled by restricting the diffusion layer. The gradients were formed on silica thin-layer chromatography (TLC) plates using organomonochlorosilanes of different sizes (C3, C4, C8, and phenyl) and visualized under UV radiation due to loss of fluorescence from the embedded fluorescent dye. Diffuse reflectance FT-IR chemically confirms the presence of C3 ligands. By constricting the diffusion of the silane vapor, the gradient shapes could be changed from concave to flat, and their lengths controlled. Photographs coupled with analysis using ImageJ were used to measure the saturation length of the gradient and the degree of curvature. C3 lengths covered 17.4–69.4% of the length of the TLC plate, and their curvature ranged from −1.9 to 11.9%, depending on the experimental conditions. Gradients formed by significantly restricting the diffusional profiles of the vapor yielded gradients with flat profiles, while those formed traditionally with no restriction of the vapor yielded gradients with an unusual concave shape due to the depletion of vapor in the center of the plate. By fitting the gradient profiles extracted from the photographs with Fick’s second law of diffusion, diffusion coefficients (*D*) could be obtained. For the C3 silane, *D* was (0.8–2.2) × 10^–3^ cm^2^/s, depending on the specifics of the configuration used. As expected, smaller values of *D* were obtained for the larger molecular weight silanes (C3 > C4 > phenyl > C8). The lower molecular weight silanes gave rise to a higher degree of silane modification, whereas the larger ones gave rise to a lesser degree of silane modification. This work shows, for the first time, how to control the size and shape of surface chemical gradients on porous supports using a vapor phase diffusion method.

## Graphical abstract

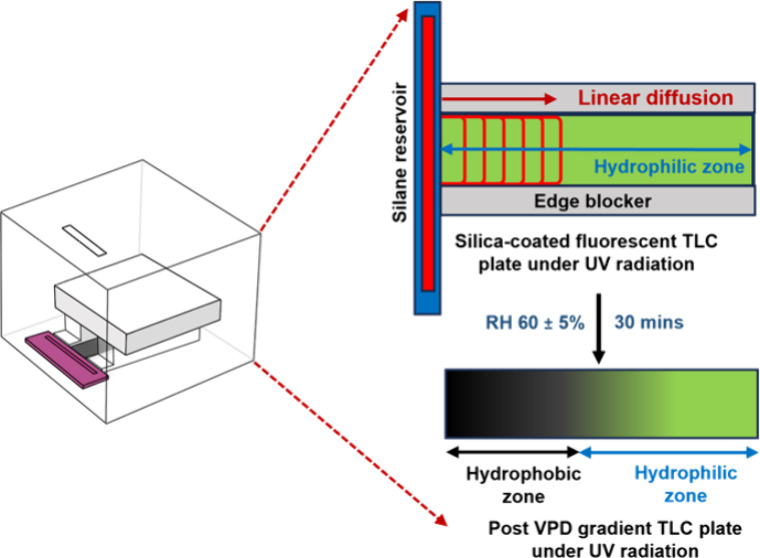

## Introduction

Chemical gradients play an important role in science.^1–4^ Concentration gradients in biological membranes direct the movement of ions, creating a gradient in potential energy.^5–7^ This potential energy can be used to transport other substances across the membrane,^8^ power cellular reactions,^9,10^ and maintain the cell’s overall homeostasis.^11,12^ In fact, these gradients are so crucial that they are considered essential sources of energy for all forms of life.^13^ Likewise, the field of electrochemistry relies on chemical gradients to direct the movement of a redox species toward the electrode interface, where reduction or oxidation can take place.^14^ When directly formed on a surface, gradient materials have served as a means to direct or guide the movement of biological cells,^15^ align DNA,^16^ study cooperativity between surface-bound functional groups,^17^ and allow water to move uphill.^18^ They have also served as high-throughput platforms to study adhesion and cell proliferation.^19,20^ These gradient materials also provide a platform for high-throughput analysis of cellular responses to different environmental conditions.^20–23^ More recently, both continuous and discontinuous stationary phase gradients have served as tools to affect chemical separations, enabling improvements in selectivity, analysis times, and resolution.^24,25^

Of the different approaches used to fabricate gradient materials,^26–29^ diffusion-based methods are particularly attractive due to their simplicity and adaptability to various substrates.^30–36^ One such method relies on the vapor-phase diffusion of a reactive, volatile compound across the length of a surface, forming a chemical gradient.^29^ In this approach, a functionalized chloro- or alkoxysilane diffuses in the gas phase from a concentrated source toward a substrate, forming a gradient as the vapor interacts with surface functional groups.^29^ Functionalized silanes, such as organoalkoxysilanes and organochlorosilanes, are particularly effective due to their volatility and rapid reactivity with Si–OH groups commonly found on surfaces, including glass and oxidized silicon wafers.^29^ In 1992, Chaudhury and Whitesides utilized this method to create wettability gradients via the diffusion of organosilanes from a silane-paraffin oil mixture.^18^ Since then, vapor phase diffusion (VPD) gradients have found diverse applications in fields such as functionalized surfaces,^37^ nanoparticle assembly,^34^ separation science,^38^ patterning surfaces,^39^ manipulation of water droplets,^40–42^ and biomolecule manipulation.^16,33,43^

One of the major challenges associated with the VPD approach is to be able to control the shape and length of the gradient and to be able to do so on a high surface area porous support. One attempt to manipulate and control the gradient steepness using the vapor deposition method was described by Efimenko and Genzer.^44^ In this work, they applied the VPD technique to flexible poly­(dimethylsiloxane) substrates, allowing fine control over gradient steepness through mechanical stretching and ultraviolet/ozone treatment. This approach enabled tunable gradients by adjusting mechanical strain and UVO exposure. Unfortunately, it is limited to flexible substrates like poly­(dimethylsiloxane), thus restricting its applicability to rigid surfaces such as silica or glass, which are commonly used. Another promising approach involves the use of a dynamic vacuum to provide more versatility in the gradient profile, particularly in conjunction with variations in the reservoir position relative to the substrate and the volatility of the silanes.^45,46^

In this article, we present a straightforward approach to modify the gradient shape from concave to flat on high-surface-area silica supports, specifically TLC plates, and investigate the variables that influence gradient shape. Unlike silicon or glass substrates, TLC plates feature a porous layer with a high surface area and a high density of silanol groups,^47^ which significantly influences the gradient formation process. They are also 3–5 times longer than the typical substrate used in VPD.^38^ Previous work has shown that vapor deposition can be used to uniformly modify a TLC plate for reversed-phase chromatography.^48^ We also recently showed that phenyl gradients can be formed on TLC plates using a modified VPD approach, and this gradient was used to separate a simple mixture.^38^ While variables such as silane concentration and relative humidity can influence the steepness of the gradient, the overall shape remains the same.^38^

The present work describes how chemical gradients with very different profiles, ranging from concave to flat, can be formed on TLC plates using n-propyldimethylchlorosilane (C3-silane) as the test silane. By restricting the diffusional profile of the vapor, a controlled and predictable modification of the gradient shape and extent can be observed. The advantage of using TLC plates with a fluorescent indicator is that the gradient shape can be easily observed under UV radiation, and detailed information can be obtained about the diffusion process. This approach is universal, simple, and works well; gradients prepared from C3, C4, C8, and phenyl silanes can easily be formed on high surface area supports. These materials thus produced have direct applicability to separation science and for the direct transport of different species from one location to another.

## Experimental Section

### Materials and Reagents

*n*-Propyldimethylchlorosilane (C3-silane, 97%), butyldimethylchlorosilane (C4-silane, 97%), octyldimethylchlorosilane (C8-silane, 97%), and phenyldimethylchlorosilane (phenyl-silane, 97%) were purchased from Gelest and used as received. Paraffin oil (light, Saybolt viscosity 130 at 100 °F) was purchased from Sigma-Aldrich, as were the TLC plates (TLC silica gel 60 F_254_). According to the manufacturer, the silica has a surface area of 480–540 m^2^/g, a pore volume of 0.74–0.84 mL/g, and a layer thickness of 210–270 μm. KBr (FT-IR grade) was purchased from Thermo Scientific and dried at 100 °C for 2 h.

### Gradient Preparation

The TLC plates were cut into 7.5 cm × 2 cm pieces and cleaned immediately before use via a plasma etcher (PE-2000, South Bay Technology Inc.; 180 mT; forward power, 24 W; reflected power, 2 W; DC bias, −239 V; 5 min). This cleaning step reduces organic contaminants and helps activate the surface through ionized gas plasma reactions, ensuring optimal surface conditions for subsequent experiments. Single-component gradients were prepared by vapor phase deposition (VPD) using one of four functionalized dimethylchlorosilanes (R–Si­(CH_3_)_2_–Cl where R = C3, C4, C8, or phenyl). The appropriate amount of silane was mixed with paraffin oil in a 50:50 ratio (unless otherwise noted), and a known volume was added into a small rectangular reservoir 3D printed from polylactic acid (PLA). The reservoir holders were of two different sizes (length × width × height): long (12 cm × 0.6 cm × 0.5 cm) and short (8 cm × 1.9 cm × 0.4 cm). The long reservoir had a silane reservoir size of (11.2 cm × 0.3 cm × 0.2 cm) and could hold up to 600 μL of sample, while the short reservoir had a reservoir of (7 cm × 0.3 cm × 0.2 cm) holding up to 380 μL of sample. To optimize the shape of the gradient, a top cover and 3D-printed edge blockers were used to control both the linear and radial silane vapor movement, as described in Results and Discussion. To form the gradient, the freshly cleaned TLC plate was placed on a flat support at a defined distance from the reservoir and centered. A Plexiglass box was placed over the top to minimize air currents. The paraffin oil/silane mixture was carefully pipetted into the reservoir through a slit in the box, and the slit was resealed. After a deposition time of 30 min, the TLC plates were removed and air-dried. The relative humidity (RH) was 60 ± 5% in the closed Plexiglass chamber.

### Gradient Characterization

The presence of a gradient on the TLC plates was assessed through a combination of visualizing the deactivation of the fluorescence from the embedded TLC plate fluorophore upon exposure to UV radiation, ImageJ analysis, and FT-IR spectroscopy. After gradient formation, the fluorescent TLC plates were placed under UV radiation and photographed at a fixed distance from the camera in a darkened box. The photograph was imported into ImageJ (Ver. 1.53e; Rasband and co-workers, National Institutes of Health, USA, https://imagej.net/ij/) for further analysis (vide infra). In the absence of modification and when under UV radiation, the TLC plates appear bright green. After modification, the TLC plate appears to saturate at the location where the gradient was formed. The mechanism for the deactivation of the fluorescence involves the hydrochloric acid released in the silanization reaction.^49^ The indicator used in TLC plates is susceptible to acid and deactivates upon exposure to it.^50^ This was experimentally confirmed by a VPD experiment using dilute HCl, as shown in Figure S1 in the Supporting Information. After exposing the TLC plate to HCl vapor for as little as 2 min, the plate appears completely saturated under UV radiation. The reactions are as follows:

$$\begin{array}{l} \mathrm{silica}\left(\mathrm{surf}\right)+\mathrm{silane}-\mathrm{Cl}\left(g\right)\to \mathrm{bound silane}+\mathrm{HCl}\left(g\right)\\ \mathrm{HCl}\left(g\right)+\mathrm{MnZn}-\mathrm{silicate fluorophore}\left(s\right)\to \mathrm{nonfluorescent products} \end{array}$$

For FT-IR analysis, a Nicolet (NEXUS 670 FT-IR) spectrometer was employed with a diffuse reflectance attachment. A portion of the powder in the gradient region of the TLC plate was removed, ground with KBr, and packed into the cell. A gold mirror served as the background. The following parameters were employed for the FT-IR study—no. of scans, 100; resolution, 4 cm^–1^; data spacing, 1.928 cm^–1^.

### Quantitative Analysis of the Gradient Profile

Once imported into ImageJ, the images were rotated as necessary using the grid tool and the rotate function to ensure that the plates were aligned vertically with minimal tilt relative to the TLC plate axes. A rectangular selection was drawn tightly around each plate to define the analysis region, and these plate images were exported as .csv files for further processing. Subsequent analysis was conducted using a custom MATLAB script. The center, left, and right profiles were plotted (the left and right profiles were extracted at 10 and 90% of the plate width), and flat regions corresponding to unmodified and highly modified areas were selected to establish baseline intensities. Regions affected by edge artifacts were excluded manually. The percent saturation length (%SL_10%_), defined as the distance between the starting point and the point where the intensity rose to 10% of the maximum value, was calculated for each profile (left, center, and right) according to the method illustrated in Figure S2. The percent curvature (%C) was determined by subtracting the center %SL_10%_ value from the average of the left and right edge %SL_10%_ values; %C provides a measure of the homogeneity of the gradient across the width of the plate.

### Diffusion Coefficient Calculations

The gradient formation process is complex, as it involves diffusion in the vapor phase, adsorption/desorption to/from the substrate, diffusion into and along the substrate, and the deactivation of the fluorescent indicator embedded in the TLC plate. The apparent diffusion coefficient could potentially be affected by all these events. In this work, we assume that vapor diffusion dominates, and we sought to keep it simple by fitting the experimental profiles to a one-dimensional diffusion model to extract the diffusion coefficients. Because the HCl deactivation of the TLC plate fluorophore is essentially a static quenching mechanism (deactivation of the fluorophor prior to excitation), the concentration of the silane in the vapor phase is assumed to be proportional to the concentration of HCl released in the silanization reaction. In this case, the Stern–Volmer relationship can be used to transform the observed fluorescence signal, *I*(*x*,*t*), obtained from the center profile, into a transformed signal *S*(*x*,*t*) that is assumed to be directly proportional to the vapor phase concentration of the silane.

1 $$S\left(x,t\right)=\frac{I\left(\infty ,t\right)}{I\left(x,t\right)}-1$$

Here, *I*(∞,*t*) is the fluorescence intensity of the TLC plate at the fully fluorescent end of the plate, and *I*(*x*,*t*) is the intensity profile across the TLC plate after 30 min VPD. This transformation allows for a direct correlation between the signal, *S*(*x*,*t*), and the concentration gradient that develops along the TLC plate. To estimate the diffusion coefficient (*D*), this signal was fit to a one-dimensional diffusion model^44,51^ in MATLAB. The signal obtained from the transformation shown in eq 1 as a function of position *x* and time *t* is fit to the equation below that uses the complementary error function (erfc),^44^

2 $$S\left(x,t\right)=S_{f}+\frac{\left(S_{i}-S_{f}\right)}{2}\mathrm{erfc}\left(\frac{x}{2\sqrt{Dt}}\right)$$

where *S*_f_ is the final signal at a far distance from the silane source and *S*_i_ is the extrapolated signal at the silane source. *D*, *S*_f_, and *S*_i_ are the parameters of the fit, where *D* is the estimated diffusion coefficient in units of cm^2^/s, and *S*_f_ accounts for the uncertainty in the meaurement of *I*(∞,*t*). Only the gradient portion of the profile was selected for fitting. The diffusion time, *t*, for each experiment was 1800 s (for a 30 min exposure). Apparent diffusion coefficients were obtained across different experimental designs and silanes, and the values ranged from 5 × 10^–5^ to 2.2 × 10^–3^ cm^2^/s, consistent with literature values for small organosilane molecules diffusing in confined systems.^37,38,52^ However, it is important to point out that these should be considered approximate values, as the processes that control gradient formation depend on many factors, including diffusion, self-assembly, reactivity and deviations from pure 1D geometry.^53^

## Results and Discussion

### C3 Gradients on TLC Plates: Initial Experiments

The VPD method is a simple approach to prepare single and multicomponent gradients on different surfaces.^29,32^ It involves placing a reactive silane, such as a functionalized chloro- or alkoxide-based silane, at a defined distance from a substrate containing reactive functional groups (Si–OH). Typically, the process takes place under a controlled environment to minimize air currents and maintain constant humidity over the deposition time.^32^ As the silane vapor moves across the substrate, it reacts with the surface silanol groups, creating a gradient in functionality. The side of the substrate closest to the vapor source has a greater extent of modification relative to the opposing end. The gradient that forms can be easily visualized when fluorescent TLC plates are used, as the added functionality deactivates the fluorescent indicator, resulting in the reduction of fluorescence (vide supra).^38^

Initial experiments began with a configuration commonly used in most VPD experiments.^32^ It consists of a rectangular reservoir with a length (*L*) holding the reactive silane, and a suitable substrate is placed at a defined separation distance (*S*) from the reservoir, all enclosed under a Plexiglass box, Figure 1A,B. The rectangular slit located on the top of the box is initially covered when it is placed over the reservoir and TLC plate. The cover is then moved to pipet the silane into the reservoir and then returned to its original position. The sample abbreviations for the different gradient plates are as follows: L*x*S*y*, where “*x*” represents the length of the reservoir in centimeters, while “*y*” represents the distance between the vapor source and the plate in millimeters. In our case, the substrate is a high surface area porous silica-coated substrate (e.g., fluorescent TLC plate; 7.5 cm × 2 cm) and the reactive silane is initially a C3 functionalized monochlorosilane, which is diluted in a 50:50 ratio with paraffin oil as an inert carrier. The use of a monochlorosilane minimizes polymerization and oligomer formation during the deposition process.

**Figure 1:**
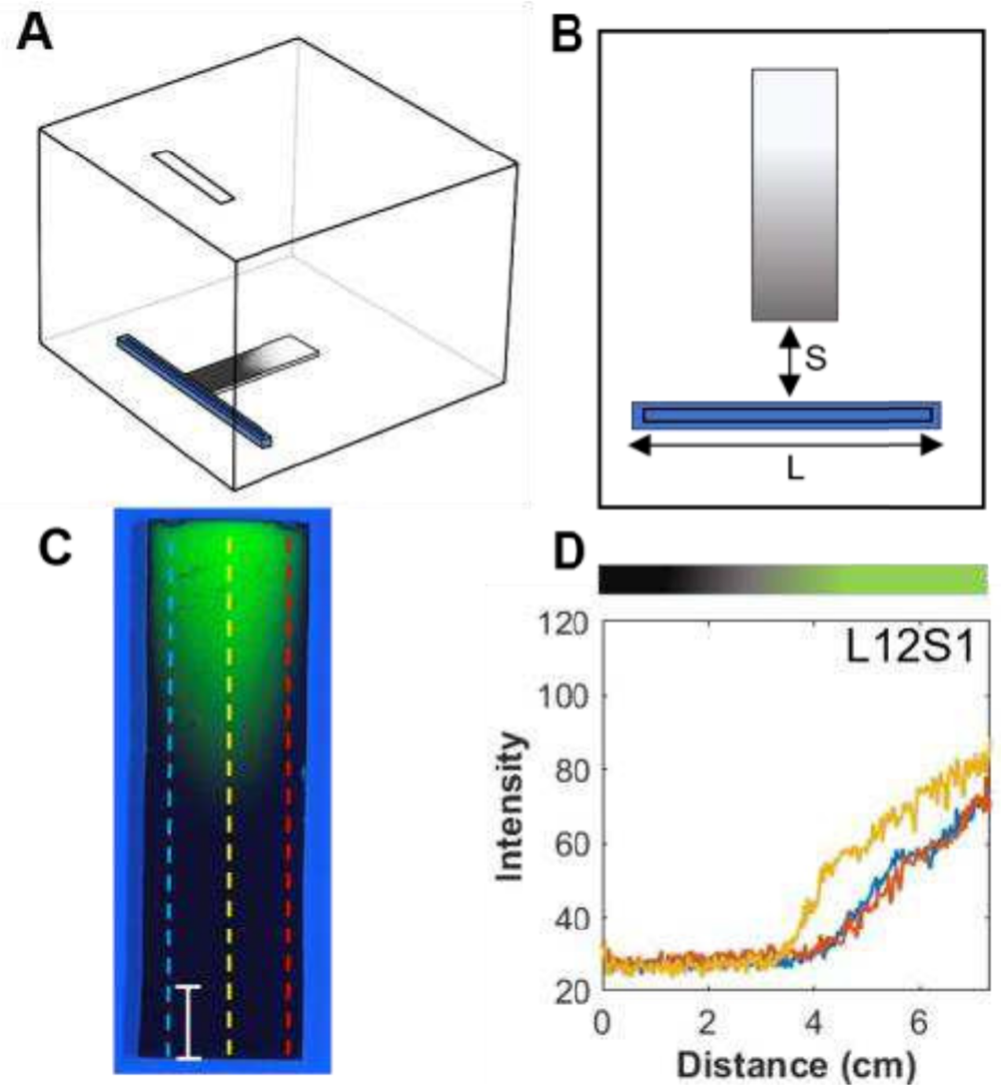
(A, B) Schematic of the VPD setup depicting the reservoir of length *L* placed a distance *S* from a TLC plate. The rectangular slit on the top of the Plexiglass box was used to dispense the silane:paraffin solution into the reservoir and was covered with a glass plate during VPD. (C) Photograph of a gradient TLC plate under UV radiation and (D) corresponding profiles at three different positions depicted by the colored dashed lines in the photograph. The distance *S* between the reservoir and the plate was 1 mm; the length *L* of the reservoir was 12 cm (L12S1). The bar represents 1 cm. These images represent experiment 1 conditions shown in Table 1.

Figure 1C shows a photograph of the TLC plate under UV radiation after gradient formation, while Figure 1D shows the longitudinal profiles of the gradient at three different positions depicted by the colored lines (center and left/right edges) for the L12S1 configuration. Under UV radiation, the gradient appears saturated as the added functionality reduces the fluorescence via deactivation of the fluorophore. What is most noticeable is the concave shape of the gradient. It is distinctly concave as indicated by the saturated periphery and the lighter central region. This shape signifies that there is greater modification at the edges of the substrate by the silane compared to the center.

To demonstrate that the TLC plate was modified with C3, diffuse reflectance FTIR was used. In this experiment, silica powder was scraped off the plate at the more modified end of the gradient and mixed with KBr. A gold mirror served as the background. Figure S3 displays the FTIR spectra from a C3 gradient TLC plate. The characteristic bands associated with silica can be seen in the 1200 to 800 cm^–1^ region.^54^ In addition, there is a broad band at ∼3400 cm^–1^ due to O–H stretching vibrations from surface silanol groups or adsorbed water. Most notably, there is a series of smaller bands near 2950 to 2850 cm^–1^, which are due to C–H stretching vibrations from the propyl (C3) groups.^48^ These can be more clearly seen in the inset. All these bands are consistent with a C3 silica-modified surface. In the absence of C3, the spectra just show the characteristic bands due to silica.

The differences in the degree of modification between the center and the edges can be more clearly seen in the profile plots shown in Figure 1D. The intensity of the fluorescent plate is low at the bottom of the gradient and gradually increases along its length. The profile obtained in the center is clearly different than those obtained at the edges, indicative of non-uniformity of the gradient along the width of the substrate. The reproducibility of gradient formation is good, as noted in the photographs of six gradient plates produced on different batches on a single day or multiple replicates on a single day, Figure S4. The gradient shapes are particularly consistent, though small differences can be noted in the length of the saturation zone.

Using the methodology described in Experimental Section, both the saturation length (%SL) and the extent of gradient curvature (%C) can be quantitatively evaluated. For the TLC plate shown in Figure 1, the %SL was 46.9%, indicating that the silane modification extends about halfway along the length of the TLC plate. The %C was 10.4%, indicating there is a fair amount of curvature. The averages for these parameters for the three plates produced in a single day (Figure S4D) using the L12S1 experimental protocol (the silane reservoir with a length of 12 cm and a separation from the TLC plate of 1 mm) were %SL 48.5 ± 1.8 and %C 11.9 ± 1.6. These values are provided in Table 1, experiment 1, along with the values for each of the subsequent experiments described in the sections that follow. For the three plates prepared on different days, %SL and %C were 44.8 ± 5.4 and 9.7 ± 0.2, respectively.

**Table 1:** Impact of Experimental Configurations on Length, Curvature, and Diffusion Coefficients

Expt no.	Exptl setup^a^	*N* ^b^	Silane	%SL^c^	%C^d^	*D* (cm^2^/s)	Comments
1	L12S1	3	C3	48.5 ± 1.8	11.9 ± 1.6	(1.07 ± 0.07) × 10^–3^	no edge blocker or top cover
2	L12S6	2	C3	32.9 ± 2.9	6.5 ± 1.7	(7.7 ± 0.3) × 10^–4^	no edge blocker or top cover
3	L8S1	2	C3	41.4 ± 1.2	7.5 ± 0.7	(1.0 ± 0.2) × 10^–3^	no edge blocker or top cover
4	L12S1	2	C3	46.5 ± 3.5	9.8 ± 2.3	(1.1 ± 0.2) × 10^–3^	no edge blocker or top cover (45°)
5	L12S1	2	C3	39.3 ± 2.2	7.3 ± 0.6	(1.6 ± 0.2) × 10^–3^	no edge blocker or top cover (90°)
6	L12S1	3	C3	69.4 ± 5.5	5.8 ± 3.7	(8 ± 3) × 10^–4^	flat edge blockers without top cover
7	L12S1	3	C3	17.4 ± 1.1	0.1 ± 0.2	(4.6 ± 0.9) × 10^–5^	flat edge blockers + top cover
8	L12S1	3	C3	64.4 ± 3.3	–1.9 ± 0.4	(2.2 ± 0.1) × 10^–3^	angled edge blockers + top cover
9	L8S1	3	C3	52.8 ± 0.6	–1.5 ± 1.1	(1.3 ± 0.1) × 10^–3^	angled edge blockers + top cover
10	L8S1	5	C4	31.5 ± 1.9	–0.4 ± 0.4	(5.6 ± 0.7) × 10^–4^	angled edge blockers + top cover
11	L8S1	4	C8	4.6 ± 0.3	1.1 ± 0.7	(9.2 ± 0.9) × 10^–5^	angled edge blockers + top cover
12	L8S1	3	phenyl	13.8 ± 1.3	1.1 ± 0.1	(3.0 ± 0.1) × 10^–4^	angled edge blockers + top cover

a *S* = separation between the reservoir and TLC plate; *L* = length of the reservoir. All angles are 0°, unless otherwise specified.

b Number of within-day replicates.

c %SL (percent saturation length) measured at the center of the plate.

d %C (percent curvature): Difference in percent saturation length between the edges and center. All ± values are standard deviations. Details of the approach for measuring percent saturation length and percent curvature are illustrated in Figure S2.

Ideally, a longitudinal gradient will be uniform across its width (e.g., homogeneous) and only non-uniform along its length. However, this is not the case. It is clear from Figure 1D that the three profiles do not overlap with each other. The gradient is longer at both edges (blue and red curves) while shorter at the center of the plate (yellow curve). In particular, at the center of the plate, the saturated portion extended to approximately 3.8 cm, while the saturated portion extended to 4.5 cm at its edges. For this unusual shape to be observed, the concentration of the reactive silane in the vapor phase must be different at the center vs. the edges of the TLC plate.

One way to understand this is to first consider that the concentration of the silane in the vapor phase is low while the density of silanol groups on the support is large. That is, the limiting reagent is the silane. As the vapor diffuses away from the source, it becomes more dilute. Eventually, in the center, the concentration of vapor-phase molecules becomes small relative to the high density of silanol groups. However, at the edges of the TLC plate, the concentration of vapor phase molecules is still relatively high because the length of the reservoir is much larger than the width of the TLC plate (>4 times wider). As a result, at the edges, the reaction between the vapor-phase silane molecules and surface silanol groups continues, ultimately resulting in this unique and unexpected concave profile. We tested this hypothesis by using a reservoir with a length (L) smaller than the width of the TLC plate; this distinct concave shape was not observed at three different deposition times ranging from 30 to 120 min, as shown in Figure S5. Only small differences in the saturation length with time were noted, which is attributed to the small volume of the reservoir and hence low concentration of silane in the vapor phase.

To better understand the deposition process and how it influences the shape of the gradient on TLC plates, both the separation distance (*S*) between the TLC plate and the reservoir and the length (*L*) of the reservoir relative to the TLC plate were varied. The most noticeable differences were expected when the distance between the plate and the reservoir changed. Increasing this separation distance should result in a shorter gradient because the vapor concentration front does not extend as far along the length of the TLC plate during the 30 min deposition period. In contrast, when this separation distance is small, the gradient will be longer because the concentrated vapor is closest to the TLC plate and can extend further along its length over the 30 min time frame. By adjusting both the separation distance and the reservoir length, it is possible to adjust the length of the gradient.

In Figure 2, photographs of two gradient TLC plates under UV radiation are shown. In one, *S* was increased to 6 mm while *L* was kept constant at 12 cm (Figure 2A), and in the other, *S* was kept at 1 mm and *L* was reduced to 8 cm (Figure 2C). In both cases, *L* was larger than the width of the substrate; the shapes are concave with differences observed in the percent saturation length (%SL) and percent curvature (%C) between the edge and the center for the different configurations, Table 1, experiments 2 and 3. Upon comparison of Figure 2A with Figure 1C, it is clear that silane modification extends further along the length of the TLC plate when S is small (e.g., 1 mm vs 6 mm), as expected. %SL is 48.5 ± 1.8 for L12S1 while it was 32.9 ± 2.9 for L12S6. Also, the uniformity across the width of the TLC plate determined from %C is smaller (e.g., more uniform) when the reservoir is placed further from the plate: 11.9 ± 1.6 vs 6.5 ± 1.7 for L12S1 and L12S6, respectively. This is expected as the relative differences in the concentration of the vapor at the center vs the edges will be less pronounced due to the longer distance (5 mm) the vapor must travel to first encounter the TLC plate. The L8S1 configuration had a %SL of 41.4 ± 1.2 and %C of 7.5 ± 0.7, while L12S1 had %SL of 48.5 ± 1.8 and %C of 11.9 ± 1.6. Collectively, these results indicate that the smaller reservoir size and the longer distance between the reservoir and the TLC plates improve gradient uniformity.

**Figure 2:**
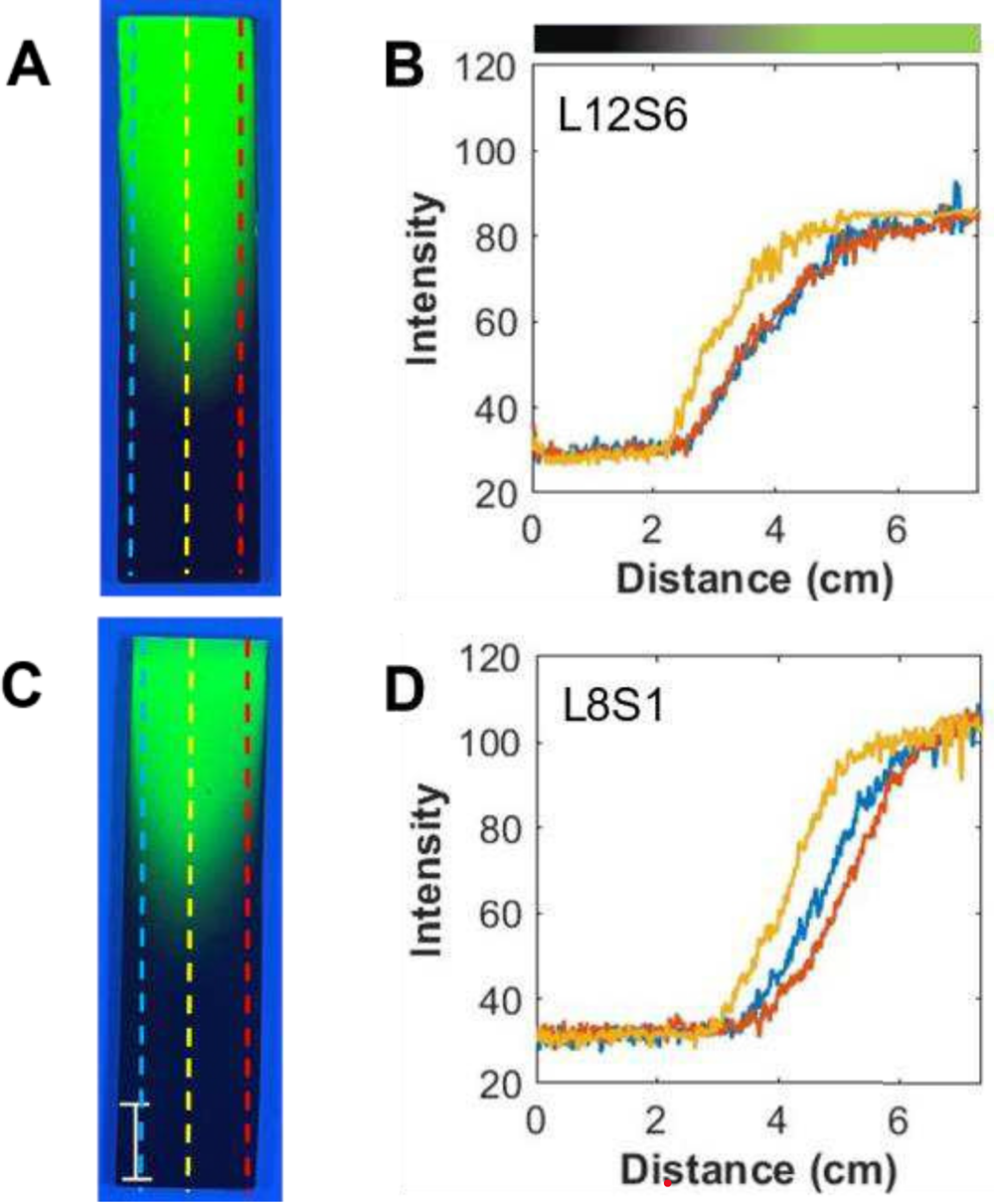
(A, C) Photographs of the C3 gradient TLC plates under UV radiation and the (B, D) corresponding profiles at three different positions (blue, red, and yellow) for L12S6 (A, B) and L8S1 (C, D). The bar represents 1 cm. These images represent experiments 2 and 3 conditions, respectively, as shown in Table 1.

As described earlier, with certain assumptions, it is also possible to extract the diffusion coefficient (*D*) from the gradient profiles. It should be noted, however, that the molecular-scale processes surrounding gradient formation and characterization are complex. In this analysis, we assume diffusion is the rate-limiting step as opposed to silane reaction kinetics or the kinetics of the HCl deactivation of the TLC fluorophore. In addition, the silane is considered to be an infinite line source, and inverse fluorescence is proportional to the silane concentration in the vapor phase. To estimate *D*, the gradient profiles are fit to eq 2 as described in Experimental Section. Note that in this approach, we are assuming that the concentration of the silane on the plate, as reflected in the inverse fluorescence, is proportional to the vapor phase concentration. The flat region closest to the silane reservoir reflects an optical saturation phenomenon, and thus, these data are not used in the fitting. Figure 3 displays the profiles obtained at the center of TLC plates, along with their fit to eq 2. The blue line in each graph represents the best fit of the experimental data to the diffusion equation. The values of *D* obtained for each of these profiles are 1.1 × 10^–3^, 1.1 × 10^–3^, and 0.75 × 10^–3^ cm^2^/s, for L12S1, L8S1, and L12S6, respectively, and the averages for the replicate plates are reported in Table 1. The trend of decreasing diffusion coefficients for these three experiments is the same as the decreasing %SL values as discussed earlier (see Table 1, experiments 1–3, for comparison). These values are within the same order of magnitude as each other and are consistent with values noted in the literature.^37,52^ This consistency between diffusion coefficient values supports that the deposition approximately follows a one-dimensional diffusion model for the three experimental setups. However, the physical and chemical processes taking place are, in actuality, much more complicated.^53^

**Figure 3:**
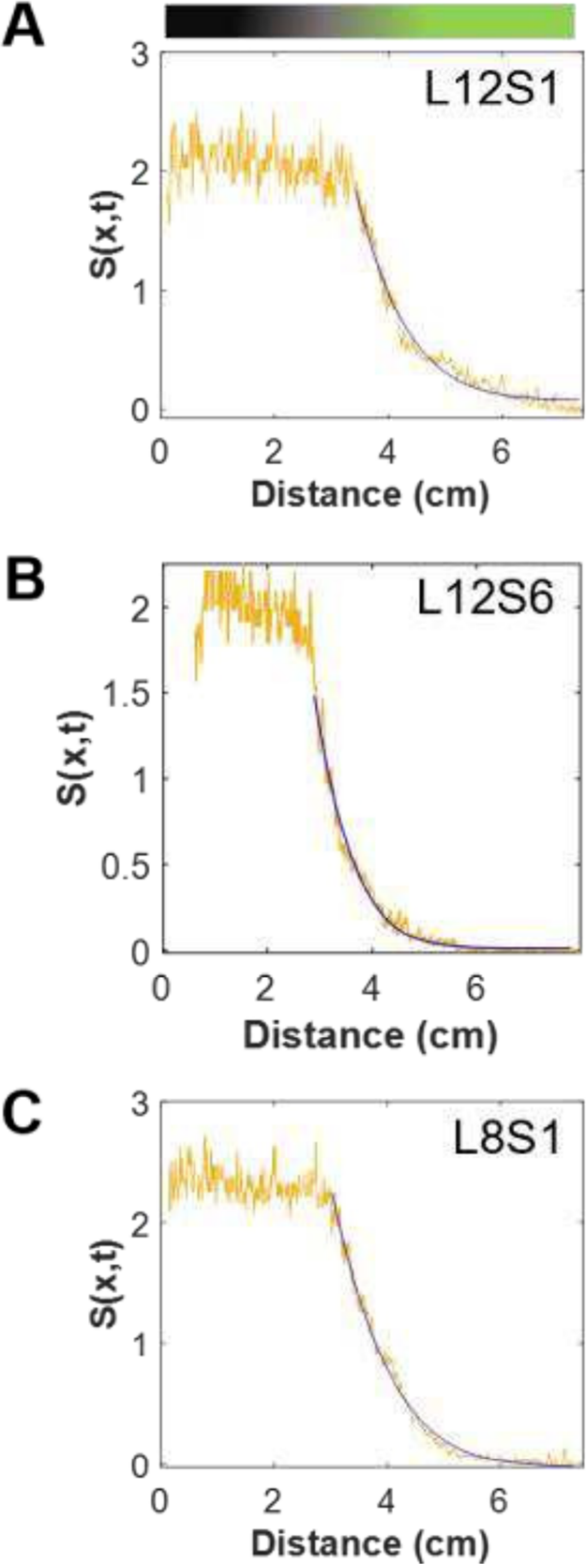
Gradient profiles of signal (eq 1) vs distance fit to a one-dimensional diffusion model described in the text. The blue line depicts the fit, and the yellow line depicts the experimental data. (A) L12S1; (B) L12S6; (C) L8S1. These plots represent experiments 1, 2, and 3 conditions, respectively, as shown in Table 1.

The next set of experiments involved placing the TLC plate at an angle relative to the vapor source, as shown in Figure 4. The results of these experiments are summarized in Table 1, experiments 4 and 5, and can be compared to the results for experiment 1 at an angle of 0°. By adjusting the angle of the TLC plate, we can see the effect on the diffusion profile of the C3 vapor and thus the shape of the gradient formed. In most VPD experiments, the angle is 0° as shown in Figures 1 and 2. The results obtained when the angles were 45 and 90° are shown in Figure 4B,C, respectively. As can be seen, the gradient profile still has the concave shape consistent with that expected with the reservoir being significantly larger than the width of the substrate. %SL values as measured from the center profile (Figure 4B,C) are 46.5 ± 3.5 and 39.3 ± 2.2, respectively, which is smaller than that obtained with the angle at 0° (Figure 1) of 48.5 ± 1.8 (see Table 1 comparisons). The orientation of the TLC plate relative to the gravitational force may influence the vapor distribution and gradient profile. When the substrate is vertical (e.g., at 90°), vapor diffusion occurs perpendicular to the gravitational vector, potentially causing a slight increase in vapor accumulation near the bottom edge due to gravitational settling. This interaction contributes to more pronounced edge effects and non-uniform gradient profiles. The deviation between the edge and the center is smaller for the gradients formed at an angle, ranging from 11.9 ± 1.6% (0°), 9.8 ± 2.3% (45°), and 7.3 ± 0.6% (90°). This indicates that plate modification is more uniform along its width when positioned at a 90° angle.

**Figure 4:**
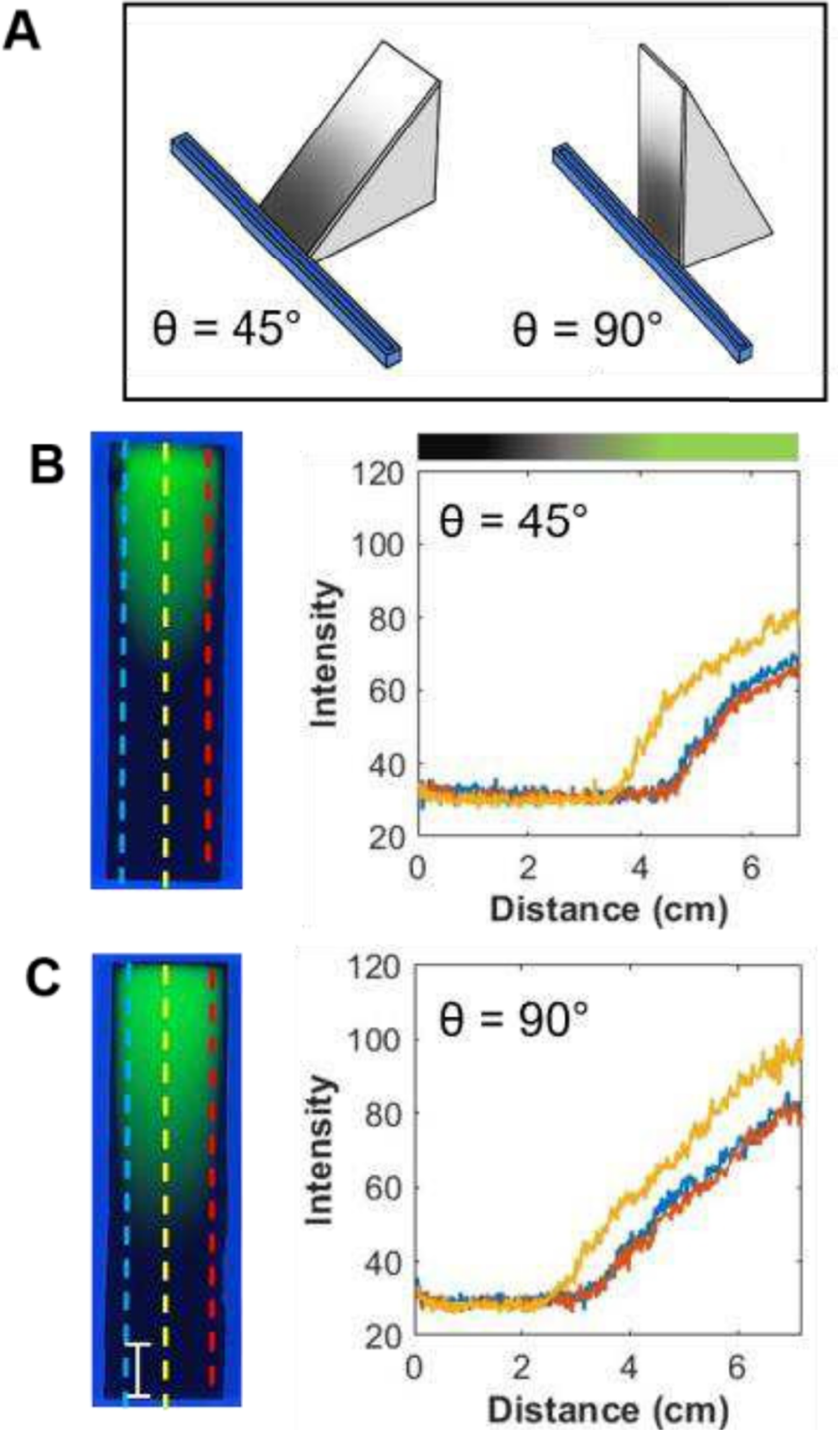
(A) Simple diagram showing the position of the vapor source reservoir relative to the TLC plate (experiments 4 and 5 in Table 1). (B, C) Photographs of two C3 gradient plates under UV-254 radiation and the corresponding profiles at three locations on the gradient plate prepared by angling them at either (B) 45° or (C) 90° relative to the vapor source. The distance between the plate and the reservoir was 1 cm, and the length of the reservoir was 12 cm (L12S1). The bar represents 1 cm.

Using the center profiles, the diffusion coefficients were obtained by fitting the profiles to eq 2, with the results illustrated in Figure S6. The values for *D* were (1.1 ± 0.2) × 10^–3^ and (1.6 ± 0.2) × 10^–3^ cm^2^/s for angles of 45 and 90°, respectively, compared to that observed when the angle is 0° ((1.07 ± 0.07) × 10^–3^ cm^2^/s). This trend is the opposite of that observed for the %SL values. This is possibly due to the %SL and *D* values reflecting differing effects of multiple processes (i.e., gravity, optical saturation, convection, and small humidity variations) rather than pure one-dimensional diffusion only.

### Restricting the Diffusion Profile

These initial results show that a C3 gradient can be easily prepared and visualized on high surface area supports such as TLC plates. Furthermore, profile analysis via ImageJ and MATLAB provides a means to quantitatively analyze the photographs and evaluate saturation length and uniformity across the width of the substrate, and calculate a diffusion coefficient. One of the challenges associated with the VPD method, however, involves controlling the shape of the gradient, particularly on TLC plates, where variations in deposition and modification along the width and the length will affect the overall performance, particularly if it is used in separations. Based on our initial results, control over the shape of the gradient can be achieved by controlling the diffusional profile of vapor-phase precursors. Henceforth, we aimed to restrict both radial and linear movement of the silane vapor by constraining the diffusion of the vapor using edge blockers that serve as walls and a top blocker that serves as a roof.

First, uniform edge blockers (height 0.5 cm) were placed on the sides of the TLC plate so that they extended 0.4 cm above the plate to better confine the vapor diffusion within the plate boundaries, as shown by the diagram on the left-hand side of Figure 5A. Figure 5B shows a photograph of the gradient thus produced along with its profiles. As can be seen, the extent of modification looks similar to that shown in Figure 2; it still has an overall concave shape, but it is obvious that the gradient extends further up the plate. %SL increases to 69.4 ± 5.5 (experiment 6, Table 1), as compared to experiment 1 (%SL of 48.5 ± 1.8). The use of edge blockers also resulted in a nearly 2-fold reduction in lateral heterogeneity (%C decreased from 11.9 ± 1.6 to 5.8 ± 3.7), demonstrating their effectiveness in improving gradient uniformity across the width of the plate.

**Figure 5:**
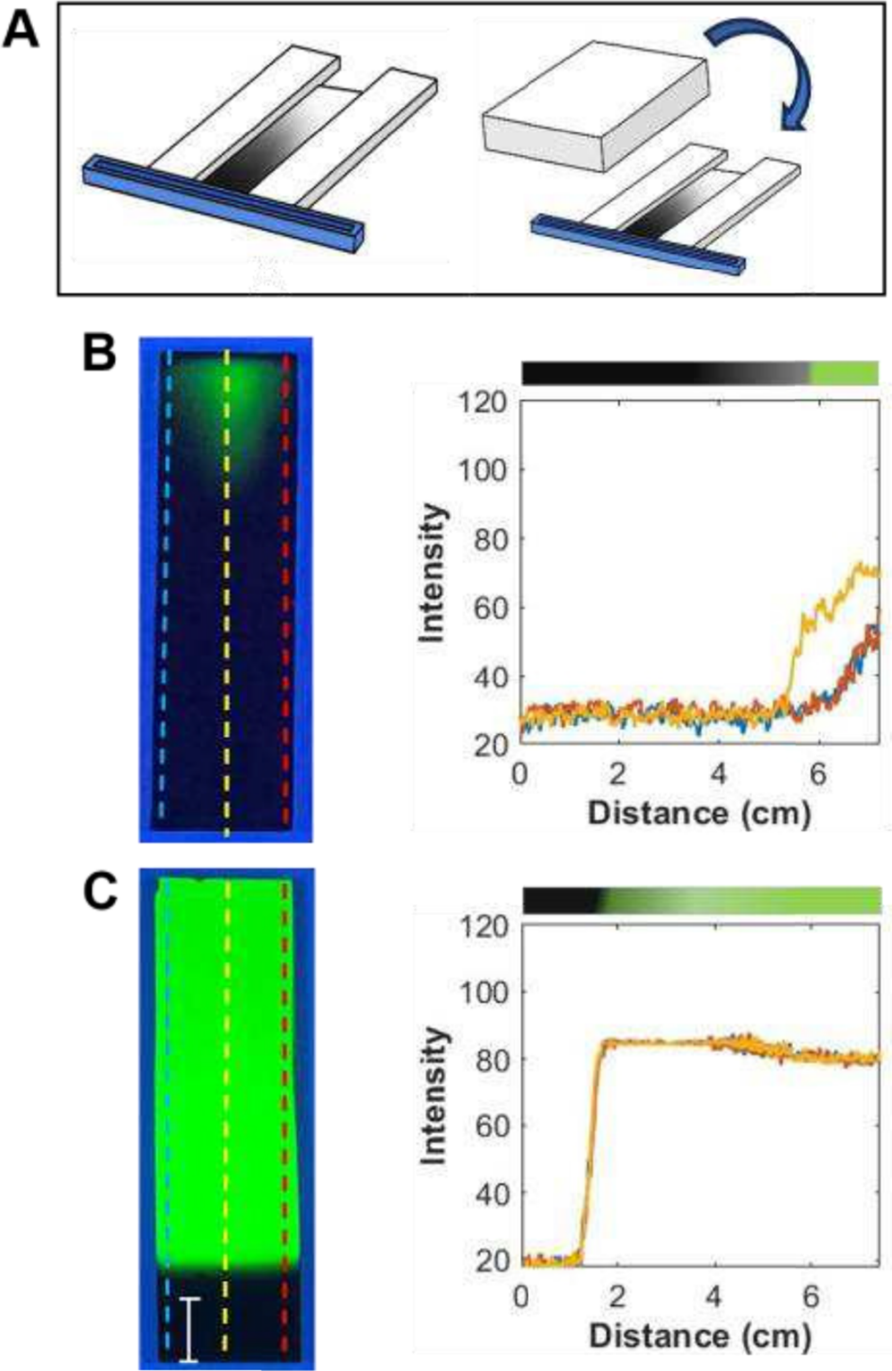
(A) Diagram of the VPD setup where either two edge blockers of the same height or two edge blockers plus a top cover are used to constrain the diffusion profile of the vapor (experiments 6 and 7 in Table 1). Photographs of the gradient plates under UV-254 radiation and corresponding profiles at three locations on the gradient plate prepared (B) without and (C) with the top cover for L12S1. The bar represents 1 cm.

To further alter the gradient shape, a top cover was also used along with the edge blockers to confine the diffusion to the location directly above the TLC plate, experiment 7 in Table 1. A diagram of this geometry is shown on the right-hand side of Figure 5A. It is important to note that the top cover was placed on the edge blockers 1.3 cm from the edge of the TLC plate. The distance between the top of the TLC slide and the bottom of the cover remained 0.4 cm. Figure 5C shows a photograph of the gradient thus produced along with its gradient profiles. As can be seen, it is very different than that shown in Figure 5B and Figure 2. Because of the placement of the top cover, the first 1.3 cm is saturated either optically or chemically. %SL was reduced to 17.4 ± 1.1, and the curvature (non-uniformity) of the gradient was eliminated (%C = 0.1 ± 0.2). Visually, the gradient is short and appears more like a step gradient. In addition, it looks uniform along the width of the TLC plate.

For both experiments, from the gradient profile, the diffusion coefficients were determined. Without the top cover, the apparent diffusion coefficient of 8 ± 3 × 10^–4^ cm^2^/s was obtained. With the top cover, a value of 4.6 ± 0.9 × 10^–5^ cm^2^/s was obtained, which is significantly lower than that obtained when there were no confinement effects. The diffusion process is only evident in the narrow 0.4 cm channel once the top cover is placed. The reason for this low diffusion coefficient is not clear at present, but it may be related to the restricted access of the vapor to the plate. Significant confinement effects will be present in this particular system, which can alter the silanization process and the dimensionality of the system. In this experiment, the volume of the space above the silica layer is quite small, and it is likely that the rate of diffusion is dominated by diffusion within the pores and between the particles, whereas in all the other experiments, the observed diffusion is likely dominated by diffusion in the volume above the silica layer.

To produce a longer gradient while maintaining homogeneity along the plate’s width, a combination of taller and slightly angled or sloped edge blockers and a top cover was used. The gap existing between the top of the TLC plate and the bottom of the cover was also increased. A side view of this arrangement is shown in Figure S7. Increasing the space between the top of the TLC plate and the bottom of the cover from 0.4 cm to 2.3–2.8 cm reduces confinement while the top cover still constrains it to a location near the surface of the TLC plate. These changes have a noticeable effect on the gradient shape, as shown in Figure 6.

**Figure 6:**
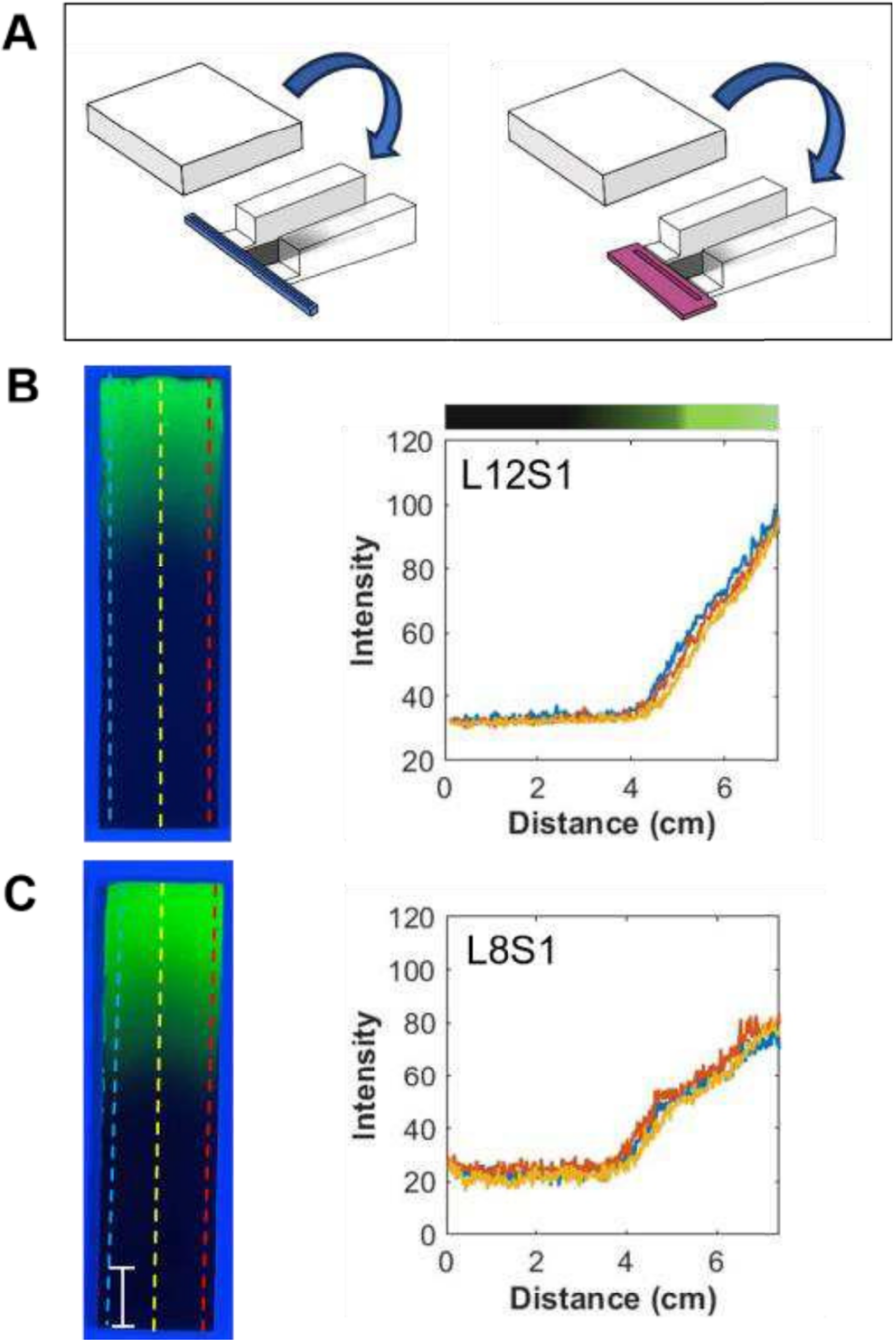
(A) Diagram of the VPD setup where two sloped edge blockers and a top cover are used to constrain the diffusion profile of the vapor (experiments 8 and 9 in Table 1). (B, C). Photographs of the gradient plates under UV-254 radiation and corresponding profiles at three locations for (B) L12S1 or (C) L8S1. The bar represents 1 cm.

For L12S1, this configuration increases %SL from 17.4 ± 1.1 (experiment 7) to 64.4 ± 3.3 (experiment 8) while maintaining uniformity along the width of the substrate (%C of −1.9 ± 0.4), as shown in Figure 6B. The three profiles overlap with very few differences observed. Similar results are observed when the reservoir is shorter (L8S1, Figure 6C, where %SL of 52.8 ± 0.6 and %C of −1.5 ± 1.1 are obtained (Table 1, experiment 9). Consistent with that observed when no edge blockers are used, the shorter reservoir produces a shorter gradient and less overall modification. The results for this experiment are summarized in Table 1, experiments 7, 8, and 9. Diffusion coefficients (*D*) obtained from the profile plots (Figure S8) support these findings with values ranging from (8 ± 3) × 10^–4^ cm^2^/s for the uniform-height edge blockers (experiment 6) without a top cover to (4.6 ± 0.9) × 10^–5^ cm^2^/s with the top cover (experiment 7), and (2.2 ± 0.1) × 10^–3^ cm^2^/s for angled edge blockers with L12S1 (experiment 8), while (1.3 ± 0.1) × 10^–3^ cm^2^/s for L8S1 (experiment 9).

### Adaptation to Other Functionalized Chlorosilanes

To evaluate the versatility of this method, other functionalized monoochlorosilanes were used as well, including phenyl, C4, and C8. These silanes have different molecular weights and vapor pressures. The molecular weights follow the trend C3 < C4 < phenyl < C8, while the boiling points, which are inversely related to vapor pressure, follow the same trend. Figure 7 shows the photographs of the functionalized TLC plates under UV radiation and the corresponding profiles obtained by extracting them using ImageJ. The data were collected using the angled edge blockers with L8S1 as shown in Figure 6. As can be seen, the gradients are homogeneous along the width of the substrate with relatively small% gradient curvature for all the silanes under these conditions. The saturation length is highly dependent on the silane. C3 shows the longest gradient as measured by %SL at 52.8 ± 0.6 (experiment 9 in Table 1), followed by C4 at 31.5 ± 1.9 (experiment 10) and phenyl-silane at 13.8 ± 1.3 (experiment 12), with C8 exhibiting the shortest degree of modification with %SL of 4.6 ± 0.3. As described earlier, the gradient profiles can be fit to eq 2 and the diffusion coefficient extracted. Figures S8 and S9 show the fitting. C3 has the highest *D* value (Figure S8D) of (1.3 ± 0.1) × 10^–3^ cm^2^/s, followed by C4 (Figure S8A) at (5.6 ± 0.7) × 10^–4^ cm^2^/s. As expected, C8 (Figure S9B) and phenyl-silane (Figure S9C) have much lower diffusion coefficients, at (9.2 ± 0.9) × 10^–5^ and (3.0 ± 0.1) × 10^–4^ cm^2^/s, respectively. This inverse relationship between molecular size and diffusion coefficient aligns with expectations, as larger molecules tend to diffuse more slowly due to their size. The diffusion coefficients confirm that smaller silanes like C3 and C4 exhibit faster diffusion and longer gradient formation, while the larger, bulkier molecules like C8 and phenyl-silane diffuse more slowly, forming shorter, more confined gradients.

**Figure 7:**
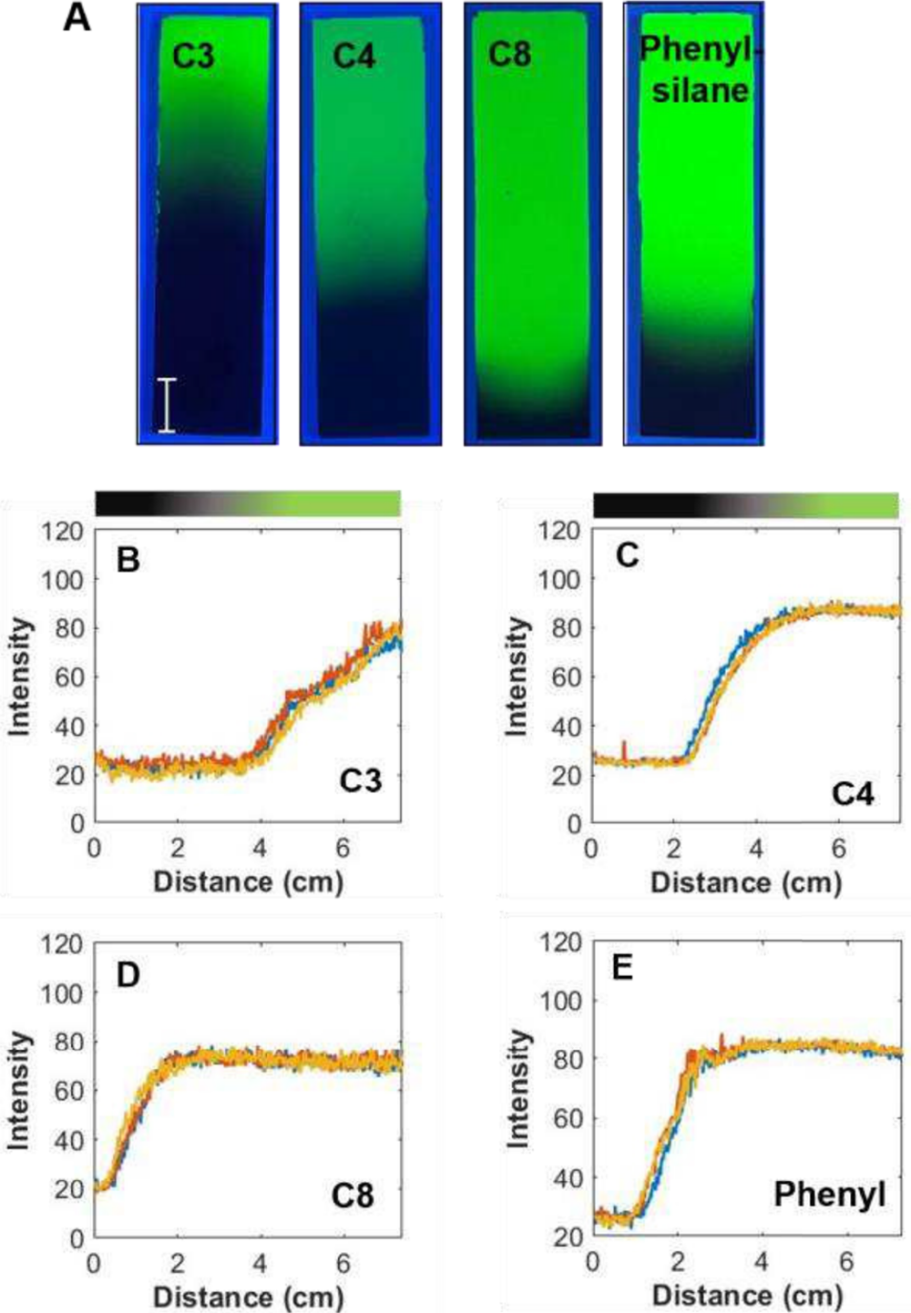
(A) Photographs of four gradient plates under UV radiation prepared using different monochlorosilanes. (B–D) Gradient profiles fit to a one-dimensional diffusion model described in the text for (B) C3, (C) C4, (D) C8, and (E) gradient plates prepared using the VPD apparatus shown in Figure 6, L8S1. These images represent experiments 9–12 conditions, respectively, as shown in Table 1.

A comparison of the predictions for the% saturation length and diffusion coefficient is shown in Figure 8. There is a correlation between the two values, but not a continuous trend, which is not too surprising, given many of the assumptions and experimental limitations. First, the %SL measurements are based on a single point where the fluorescent intensity is at 10% of its maximum value. In contrast, the diffusion coefficients are based on the region between the minimum and maximum fluorescence intensity (see fitted curves in Figures 3, S6, S8, and S9). Second, we have no information from these imaging experiments for the part of the plate closest to the vapor source, due to the complete saturation of fluorescence. Additionally, while we have assumed a one-dimensional diffusion model, none of the experiments really meet this requirement.

**Figure 8:**
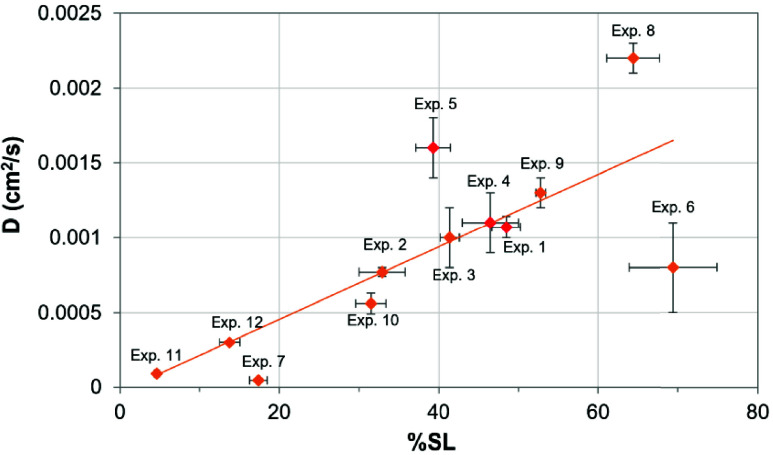
Plot of diffusion coefficient (*D*) vs percent saturation length (%SL) for various vapor phase diffusion (VPD) configurations. Error bars represent standard deviations for both *D* and %SL.

Despite these limitations, we can see several trends. First the series of experiments where lateral diffusion is blocked with angled blockers and a top cover is used for the various silanes (C3, C4, phenyl and C8, experiments 9–12 in Table 1) show a consistent trend as indicated by the orange trend line in Figure 8. The sequence of experiments where the angle of the TLC plate relative to the reservoir varies from 0° to 45° to 90° is shown in red (experiments 1, 4, and 5 using the L12S1 configuration with no edge blocker or top cover). In this case, the apparent diffusion coefficient increases with increasing angle, but the%SL decreases with increasing angle. It is not clear what the reason is for this trend. Also, it is clear when comparing similar experiments, both with and without a top cover (i.e., experiments 7 and 6 for the flat edge blockers), that the presence of the top cover decreases the saturation length.

## Conclusion

VPD methods provide a simple and easy approach to form gradient materials from a wide variety of functionalized silanes. However, one of the important challenges has been to be able to control and modify the shape of the gradient along its width and length. Homogeneity along the width of the substrate is as important as the heterogeneity along its length. For chromatography or directed transport applications, uniformity along the width of the substrate will impact the reproducibility. For investigations related to understanding molecular-scale environments, heterogeneity along both the width and length of a substrate may be better. This work lays the groundwork for understanding how to control the profile of a gradient by controlling the vapor phase profile. Key parameters influencing gradient formation include the relative size difference between the reservoir and the width of the substrate, reservoir design itself, the orientation angle, and the use of inert blockers to constrain the vapor phase near the substrate surface. The manipulation of these factors enabled control over silane diffusion and thus gradient profiles from linear to concave. For example, longer reservoirs enhanced silane modification and extended saturation lengths with a distinct concave shape, while shorter reservoirs facilitated more uniform coverage along the width of a substrate with a slightly shorter saturation length. By confining the diffusion layer near the reactive substrate, short, steep gradients with excellent homogeneity in width can be obtained. The use of fluorescent TLC plates as substrates offers a straightforward and visual means to assess gradient formation and reproducibility. Intensity profiles extracted from UV-illuminated photographs can be used to estimate apparent diffusion coefficients that characterize silane transport in the vapor phase. These values reflect, in part, how vapor-phase diffusion governs the spatial formation of the surface-bound gradient. Overall, this study highlights the versatility of VPD methods in controlling gradient profiles on substrates by optimizing reservoir design, orientation, and diffusion confinement, offering valuable insights into tailoring silane diffusion for applications ranging from molecular-scale investigations to surface property modulation. Future studies will be undertaken to more quantitatively understand the extent to which the silane vapor modifies the silica, particularly along its thickness. Over the long-term, and with an improved understanding of gradient formation on porous supports, these materials may have possible applications in chromatographic separations, as a high-throughput tool to optimize the reaction environment of catalytic sites on a porous catalytic support such as those prepared from acidic/basic functional groups, and/or controlling mass transport within a porous material such as through the use of fluorinated silanes.

## Supplementary Material



## Notes

Additional figures that describe the process for calculating saturation length and curvature and show the FTIR spectrum of a gradient modified TLC plate, replicate photographs of the gradient TLC plates under UV radiation, a different VPD setup, gradient profiles fit to a one-dimensional diffusion model, and a side view with dimensions of the angled edge blocker used to confine the diffusion layer (PDF)
