# Supplementary material for: Controlling the Diffusion Profile in the Vapor Phase Deposition of Silanes for Gradient Fabrication
Source: Langmuir. 2026 Mar 26;42(13):8983–93. doi: 10.1021/acs.langmuir.5c03462 (PMC13063807; doi:10.1021/acs.langmuir.5c03462)
Supplement: Supplementary file 1 [file la5c03462_si_001.pdf]

## Supplemental Information

# Controlling the diffusion profile in the vapor phase deposition of silanes for gradient fabrication

Shomaly Chakraborty, Ash Young, Md. Abdullah Al Macktuf, Sarah C. Rutan*, Maryanne M. Collinson*
Department of Chemistry, Virginia Commonwealth University, Richmond, Virginia 23284, United States.

*Corresponding authors, mmcollinson@vcu.edu; srutan@vcu.edu

**Table of Contents:**

**Figure S1.** Photographs of TLC plates under UV radiation after exposure to HCl

**Figure S2.** Stepwise process for calculating gradient length and curvature.

**Figure S3.** Diffuse Reflectance FTIR spectrum collected from the highly modified end of a C3 gradient plate

**Figure S4.** Photographs of gradient TLC plate replicates under UV radiation demonstrating reproducibility

**Figure S5.** VPD diagram and photograph acquired under UV radiation for the case where the length of the reservoir is smaller than the width of the TLC plate for different deposition times.

**Figure S6.** Gradient profiles fit to a one-dimensional diffusion model

**Figure S7.** Side view of the angled edge blocker used to confine the diffusion layer

**Figure S8.** Gradient profiles fit to a one-dimensional diffusion model for gradients prepared using different experimental setups

**Figures S9.** Gradient profiles fit to a one-dimensional diffusion model for gradients made with C4, phenyl, and C8 silanes

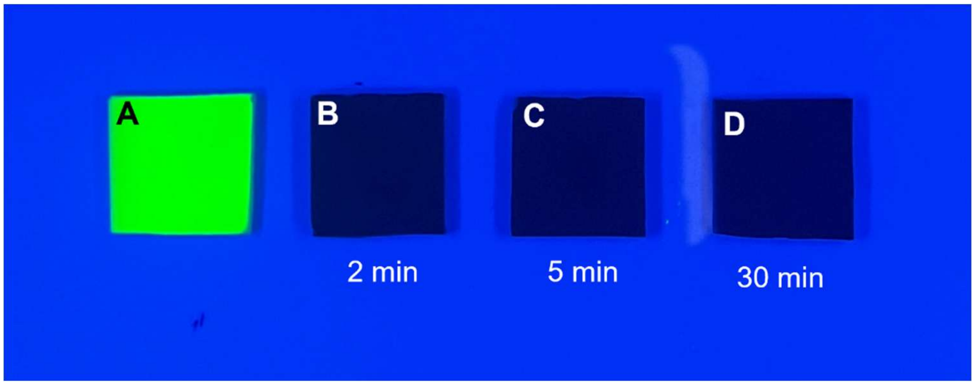


**Figure S1.** Photographs of unmodified TLC plates under UV radiation after exposure to (A) water vapor and (B, C, D) concentrated HCl vapor for (B) 2, (C) 5, and (D) 30 min.

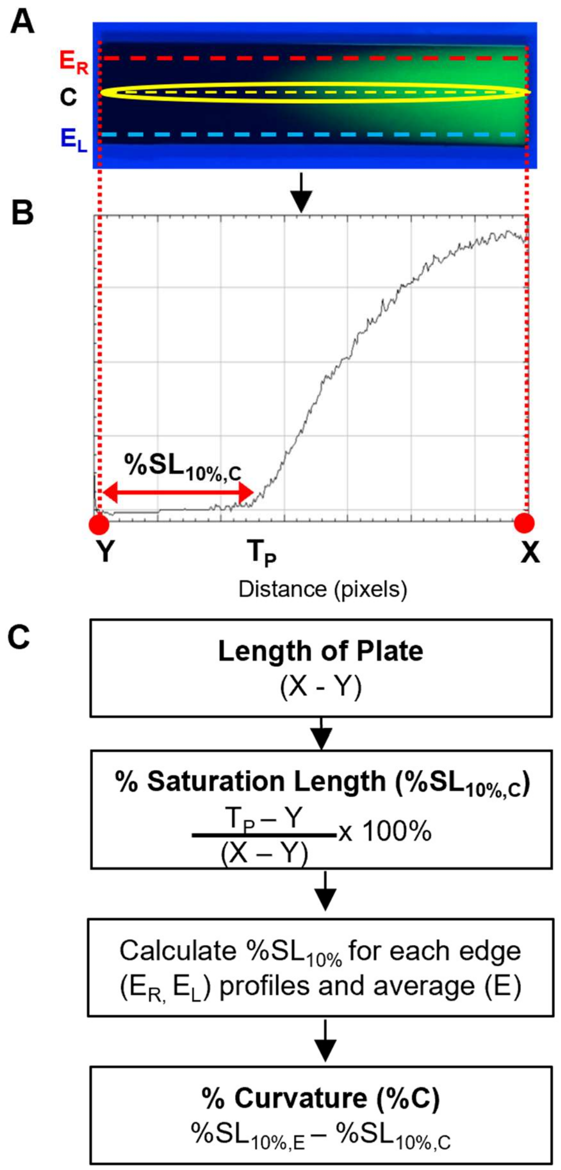


**Figure S2.** Step-by-step process for evaluating the saturation length and curvature. A) Photograph of TLC plate under UV radiation. B) Profile obtained from the center of the TLC plate image. C) Calculations. $T_p$ = 10% intensity point, $E_R$ = right edge, $E_L$ = left edge, E = avg. of right and left edge, C = center.

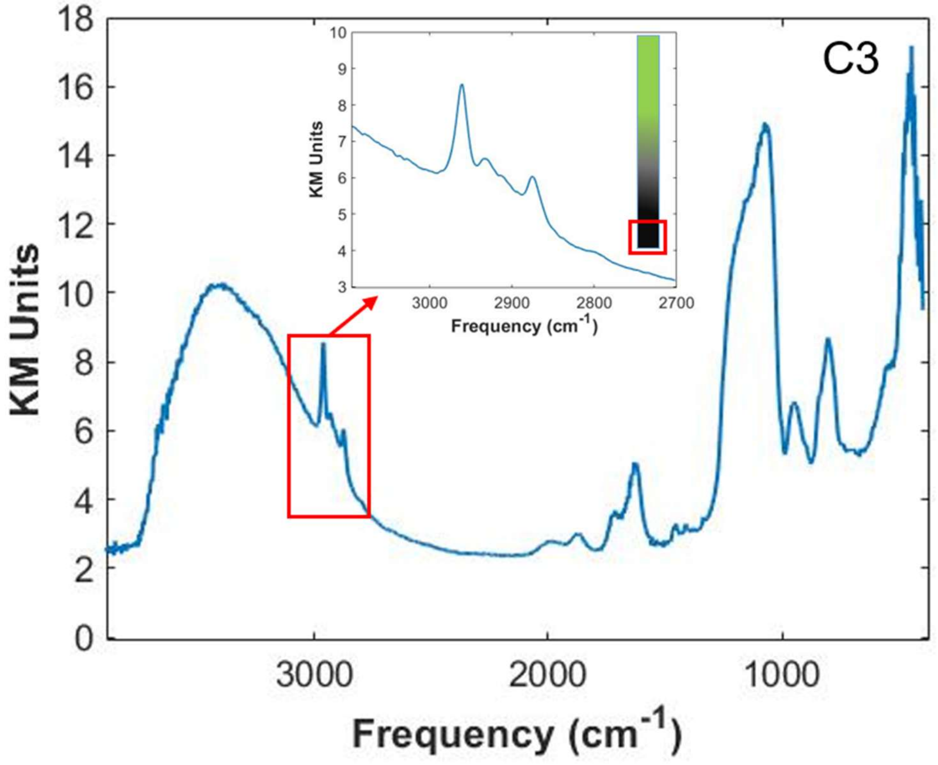


**Figure S3.** Diffuse Reflectance FT-IR spectrum collected on a C3 gradient TLC plate. Inset: Expanded view of the spectrum over the 3100-2700 $cm^{-1}$ range. A gold mirror served as the background.

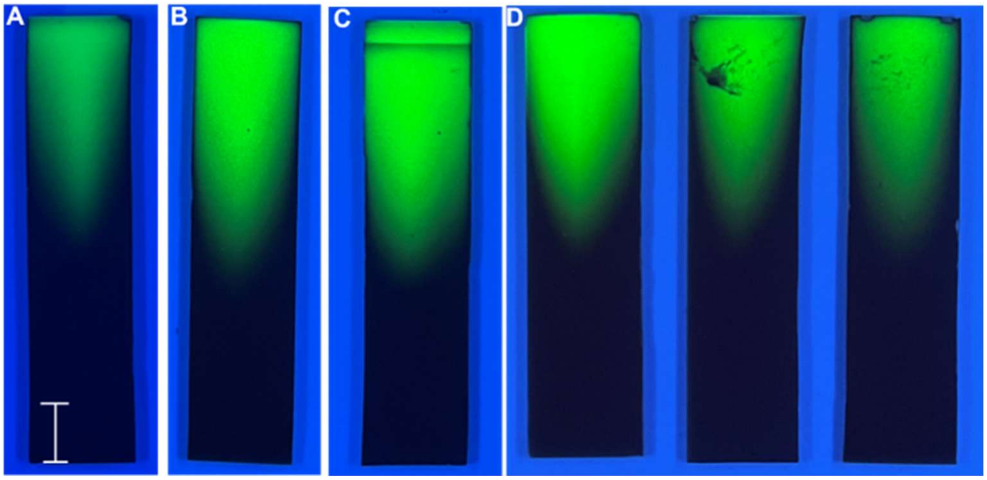


**Figure S4.** Photographs of the gradient TLC plate replicates under UV radiation with L12S1 demonstrating reproducibility. (A-C) show consistent results across different batches, while (D) highlights reproducibility within a single day. These images represent experiment 1 conditions shown in Table 1. The bar represents 1 cm.

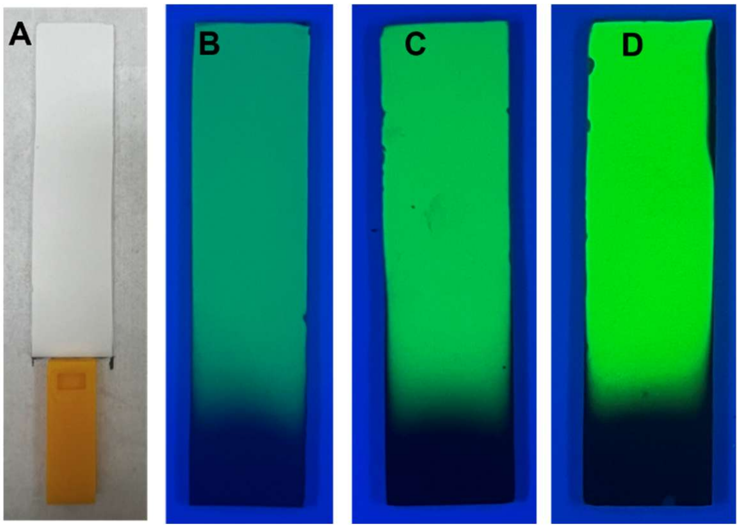


**Figure S5.** (A) VPD experimental set-up using a reservoir (dark orange) that is narrower (0.4 cm x 0.7 cm x 0.2 cm) than the width of the plate. The resultant gradient plate under UV radiation after VPD for (B) 30 min, (C) 60 min, and (D) 120 min.

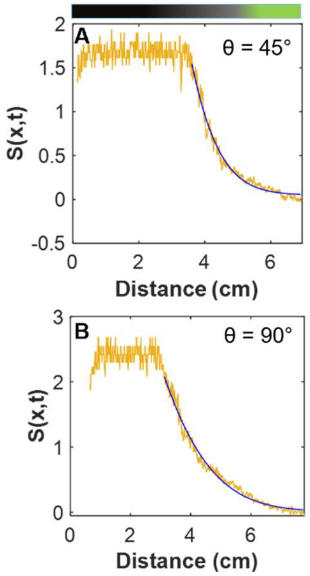


**Figure S6**. Gradient profiles (signal (eq. (1) vs. distance) fit to a one-dimensional diffusion model described in the text. The blue line depicts the fit, and the yellow line depicts the experimental data. The experimental setup is L12S1, where the TLC plate was oriented either at (A) 45° or (B) 90° as shown in Figure 4. These images represent experiments 4 and 5 conditions, respectively, as shown in Table 1.

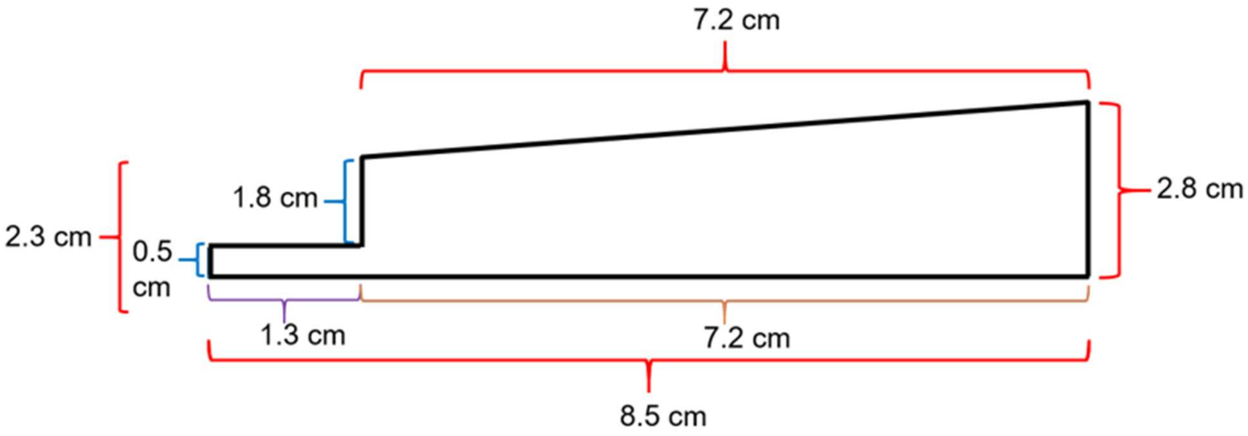


**Figure S7**. Side view of the angled edge blocker used in experiments 8-12 in Table 1.

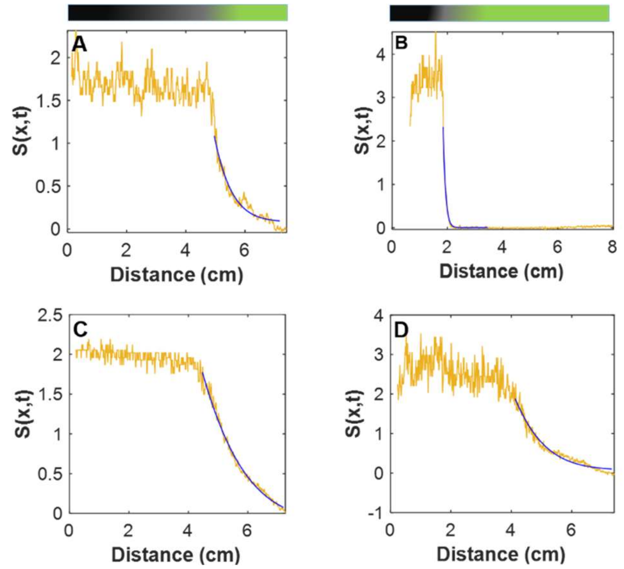


**Figure S8**. Gradient profiles (signal (eq. (1) vs. distance) fit to a one-dimensional diffusion model described in the text. The blue line depicts the fit, and the yellow line depicts the experimental data. The reservoir configuration is L8S1, where the setup configurations are (A) flat edge blocker without top cover, (B) flat edge blocker with top cover, (C, D) sloped edge blocker with either (C) L=12 cm (L12S1) or (D) L=8 cm (L8S1). These plots represent experiments 6, 7, 8, and 9 conditions, respectively, as shown in Table 1.

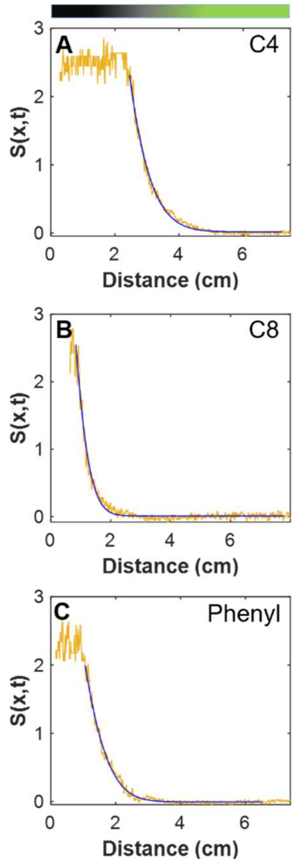


**Figure S9**. Gradient profiles (signal (eq. (1) vs distance) fit to a one-dimensional diffusion model described in the text. The blue line depicts the fit, and the yellow line depicts the experimental data. The reservoir configuration is L8S1. These images represent experiment 10, 11, and 12 conditions, respectively, as shown in Table 1.
